# Removal of large middle molecules via haemodialysis with medium cut-off membranes at lower blood flow rates: an observational prospective study

**DOI:** 10.1186/s12882-019-1669-3

**Published:** 2019-12-31

**Authors:** Tae Hoon Kim, Seok-hyung Kim, Tae Yeon Kim, Hae Yeul Park, Kwon Soo Jung, Moon Hyoung Lee, Jong Hyun Jhee, Jung Eun Lee, Hoon Young Choi, Hyeong Cheon Park

**Affiliations:** 1 Department of Internal Medicine, Yongin Severance Hospital, Yonsei University College of Medicine, Yongin-si, Gyeonggi-do 17046 Republic of Korea; 2 Department of Internal Medicine, Chuncheon Sacred Heart Hospital, Hallym University College of Medicine, Chuncheon-si, Gangwon-do 24253 Republic of Korea; 3 Department of Internal Medicine, Gangnam Severance Hospital, Yonsei University College of Medicine, 211 Eonju-ro, Gangnam-gu, Seoul, 06273 Republic of Korea; 4 Severance Institute for Vascular and Metabolic Research, Yonsei University College of Medicine, Seoul, 03722 Republic of Korea

**Keywords:** Medium cut-off membrane, Haemodialysis, Predilution online haemodiafiltration, Large middle molecules, Uraemic toxins

## Abstract

**Background:**

Online haemodiafiltration (OL-HDF) may improve middle molecular clearance in contrast to conventional haemodialysis (HD). However, OL-HDF requires higher convective flows and cannot sufficiently remove large middle molecules. This study evaluated the efficacy of a medium cut-off (MCO) dialyser in removing large middle molecular uraemic toxins and compared it with that of conventional high-flux (HF) dialysers in HD and predilution OL-HDF.

**Methods:**

Six clinically stable HD patients without residual renal function were investigated. Dialyser and treatment efficacies were examined during a single midweek treatment in three consecutive periods: 1) conventional HD using an HF dialyser, 2) OL-HDF using the same HF dialyser, and 3) conventional HD using an MCO dialyser. Treatment efficacy was assessed by calculating the reduction ratio (RR) for β2-microglobulin (β2M), myoglobin, κ and λ free light chains (FLCs), and fibroblast growth factor (FGF)-23 and measuring clearance for FLCs.

**Results:**

All three treatments showed comparable RRs for urea, phosphate, creatinine, and uric acid. MCO HD showed greater RRs for myoglobin and λFLC than did HF HD and predilution OL-HDF (myoglobin: 63.1 ± 5.3% vs. 43.5 ± 8.9% and 49.8 ± 7.3%; λFLC: 43.2 ± 5.6% vs. 26.8 ± 4.4% and 33.0 ± 9.2%, respectively; *P* <  0.001). Conversely, predilution OL-HDF showed the greatest RR for β2M, whereas MCO HD and HF HD showed comparable RRs for β2M (predilution OL-HDF vs. MCO HD: 80.1 ± 4.9% vs. 72.6 ± 3.8%, *P* = 0.01). There was no significant difference among MCO HD, HF HD, and predilution OL-HDF in the RRs for κFLC (63.2 ± 6.0%, 53.6 ± 15.5%, and 61.5 ± 7.0%, respectively; *P* = 0.37), and FGF-23 (55.5 ± 20.3%, 34.6 ± 13.1%, and 35.8 ± 23.2%, respectively; *P* = 0.13). Notably, MCO HD showed improved clearances for FLCs when compared to HF HD or OL-HDF.

**Conclusions:**

MCO HD showed significantly greater RR of large middle molecules and achieved improved clearance for FLCs than conventional HD and OL-HDF, without the need for large convection volumes or high blood flow rates. This would pose as an advantage for elderly HD patients with poor vascular access and HD patients without access to OL-HDF.

**Trial registration:**

Clinical Research Information Service (CRIS): KCT 0003009. The trial was prospectively registered on the 21 Jul 2018.

## Background

Patients with end-stage renal disease (ESRD) have higher rates of cardiovascular (CV) morbidity and mortality than the general population. In addition to traditional risk factors, haemodialysis (HD) patients have a higher prevalence of non-traditional risk factors, such as anaemia, inflammation, oxidative stress, and accumulation of toxins that are inherent to the ‘uraemic milieu’ [1]. Middle molecules have a broad range of molecular size from 500 to 60 kDa and include a number of cytokines, adipokines, growth factors, and other signalling proteins that are significantly elevated in dialysis patients compared with those in individuals with normal kidney function. The serum levels of interleukin (IL) 1β (17.5 kDa), IL-6 (21.0–28.0 kDa), and IL-18 (18.0 kDa) as well as κ (22.5 kDa) and λ (45.0 kDa) free light chains (FLCs) are also elevated in patients with advanced chronic kidney disease (CKD) [2]. The levels of fibroblast growth factor (FGF)-23 (22.5–32.0 kDa), a growth factor involved in renal phosphate handling and the development of mineral and bone disorders in CKD, also can increase up to > 200-fold. These and many other middle molecules are implicated in chronic inflammation, atherosclerosis, structural cardiac disease, and protein-energy wasting and are key players in the inflammation-CVD pathway [3].

The introduction of HDF with online production of large volumes of substitution fluid (online HDF [OL-HDF]) markedly enhanced convective removal of middle molecules in contrast to that with high flux (HF) HD. Large observational cohort studies suggest that OL-HDF treatment may decrease mortality risk compared to conventional HD [4]. However, primary analysis of recent randomized controlled trials failed to demonstrate definite survival benefit of OL-HDF compared with conventional HD [5–7]. Current HF membranes have cut-off size values of approximately 20 kDa, and thus, have a limited ability to clear larger middle molecules such as serum FLC and FGF-23. Therefore, OL-HDF with maximal convection volumes may still be insufficient to prevent accumulation of these large uraemic toxins that accelerate the development of CVD in HD patients [8, 9].

The so-called medium cut-off (MCO) membrane has a steep sieving curve characterised by high membrane cut-off and high retention onset values that are close to but lower than those of albumin [10, 11]. Such features enable MCO membranes to enhance the removal of a wide range of large middle molecules approximately up to 50.0 kDa with minimal albumin loss. Only a few studies have compared the efficacy between HD with MCO membranes and postdilution OL-HDF with conventional HF membranes [12, 13]. HD treatments using MCO membranes effectively removed a wide range of middle molecules in contrast to HF HD and even surpassed the performance of postdilution OL-HDF for large middle molecules. However, comparisons between HD with MCO membranes and predilution OL-HDF in the removal of middle to large uraemic toxins in Asian HD patients are lacking. The current study aimed to investigate the reduction ratios (RR) of an MCO membrane in the removal of middle molecules and whether there are differences in the RR of large uraemic solutes between HD with MCO membranes and conventional HF HD or predilution OL-HDF in actual clinical settings.

## Methods

### Study population

This observational prospective study was performed at the dialysis unit of Gangnam Severance Hospital, Seoul, Republic of Korea and approved by its Institutional Review Board (No. 3–2018-0151, KCT 0003009). We collected clinical and HD treatment data from six clinically stable HD patients who provided their written informed consent.

### Study design

Treatment efficacies were examined during a single midweek treatment in three consecutive periods with a 2-week washout period: 1) conventional HD using an HF membrane (HF HD; Rexeed-21A®, Asahi Kasei Medical, Tokyo, Japan), 2) OL-HDF using the same HF membrane in predilution mode (predilution OL-HDF), and 3) conventional HD using an MCO membrane (MCO HD; Theranova 400®, Baxter, Hechingen, Germany). The HD membrane characteristics are described in detail in Table 1.

**Table 1:** Characteristics of the dialysers

Dialyser	Rexeed-21A®	Theranova 400®
Membrane material	PS	PAES/PVP blend
Surface area (m^2^)	2.1	1.7
Membrane wall thickness (μm)	45	35
Membrane inner diameter (μm)	185	180
Flux	HF	MCO
β2M (11.8 kD)	0.85	1
Albumin (66.5 kD)	0.002	0.008
UF coefficient (mL/h/mmHg)	90	48
KoA urea (mL/min^2^)	1569	1482

*PS* polysulfone, *PAES* polyarylethersulfone, *PVP* polyvinylpyrrolidone, *UF* ultrafiltration, *HF* high-flux, *MCO* membrane cut-off, *KoA* mass transfer area coefficient

The dialysis prescriptions were based on their routine prescription. The dialysis session duration was 4 h, and the BFR was 250 mL/min, with the ultrafiltration volume adjusted according to each patient’s dry weight (unchanged from their usual treatment). OL-HDF was performed in predilution mode based on each patient’s usual total convective ultrafiltration volume.

The efficacy of each dialyser membrane treatment was assessed by calculating the RR for the small and middle molecular uraemic toxins: urea (60 Da), phosphate (95 Da), creatinine (113 Da), uric acid (168 Da), β2M, myoglobin (16.7 kDa), κFLC, λFLC, and FGF-23.

Blood samples were collected prior to the beginning and at the end of each HD session. Postdialysis blood samples were obtained 20 s after diminishing the pump speed to 50 mL/min. We calculated the RR for the small and large middle molecules using the following formula: RR (%) = [1 − (Cpost/Cpre)] × 100, where Cpre and Cpost are the measured plasma levels of the solute before and after dialysis, respectively. The postdialysis levels were corrected for haemoconcentration by assuming that the distribution volume of each free surrogate large middle molecule is equal to the extracellular volume, that the extracellular volume is 20% of the end dialysis body weight, and that intradialytic body weight loss reflects the change within the extracellular volume. Thus, the postdialysis large middle molecular levels were corrected by dividing the raw data of the large middle molecules by [1 + (intradialytic weight loss [kg])/0.2 (end dialysis body weight [kg])] [14]. The postdialysis albumin level was corrected using the haematocrit level [15]. Spent dialysate was collected continuously at 10 mL/min through the dialysate drain. Overall clearance was calculated by dividing the total FLC removal by the area under the plasma water concentration–time curve. Total FLC removal was calculated by multiplying the dialysate FLC concentration by the ultrafiltration volume and the total spent dialysate volume [16].

### Measurement of the uraemic toxins and albumin levels

The levels of β2M and FLC were determined using the commercially available equipment Immulite 2000 XPi (Siemens Healthcare Diagnostics SA, Zürich, Switzerland; reference interval, 0.61–2.37 μg/mL) and SPA Plus® (Binding Site, Birmingham, UK; reference interval, 5.71–26.30 mg/L). The level of FGF-23 was estimated via enzymatic measurement using the FGF-23 (C-terminal) ELISA kit (Biomedica, Vienna, Austria). The albumin levels in the spent dialysates were assessed using the albumin ELISA kit (ab227933; Abcam, Cambridge, MA, USA). All serum, plasma, and spent dialysate samples were collected and sent to laboratory facilities under standardised conditions.

### Statistical analysis

Data were expressed as numbers (percentages) and means ± standard deviations or medians (interquartile ranges) according to the presence of normal distribution. The variables from the six patients were classified into three different groups: HF HD, predilution OL-HDF, and MCO HD; these were calculated using the linear mixed model for unstructured covariance patterns. The Friedman test, a nonparametric test, was used in accordance with non-normality of the pre- and post-κFLC parameters. Post hoc *P* values, which reflect the significance of the difference between each pair determined by the least significant difference, were used. For more conservative interpretation, *P* values of < 0.0167 (Bonferroni method) were considered statistically significant. Analyses were performed using the SAS version 9.3 (SAC Institute Inc., Cary, NC, USA).

## Results

### Clinical features of the patients

Six clinically stable HD patients with no residual renal function participated in this observational prospective study. Their baseline clinical characteristics are summarised in Table 2. They were all elderly male patients (age, 66.1 ± 9.1 years) and had a mean HD vintage of 3.8 ± 1.8 years. The mean dry weight was 64.0 ± 8.3 kg, and the mean UF during predilution OL-HDF treatment was 1850.0 ± 634.8 mL with no significant differences among 3 treatment modalities (*P* = 0.75). OL-HDF was performed in predilution mode and delivered sufficient convection volumes (49.91 ± 0.47 L/session).

**Table 2:** Baseline characteristics of the study population

Variables	Mean ± SD or N (%)
Age, years	66.1 ± 9.1
Male sex	6 (100.0)
Vascular access	
Native, graft	5 (83.3), 1 (16.7)
Cause of ESRD	
ADPKD, HTN, diabetes	2 (33.3), 1 (16.6), 3 (50.0)
Height, cm	171.6 ± 4.9
Dry weight, kg	64.0 ± 8.3
Volume distribution, L	36.3 ± 3.4
Urea reduction ratio, %	72.3 ± 2.5
UF volume, mL	
High-flux HD	1833.3 ± 585.4
Predilution OL-HDF	1850.0 ± 634.8
MCO HD	2033.3 ± 436.7
Dialysis vintage, years	3.8 ± 1.8
Residual urine level, L	0.0 ± 0.0
CRP level, mg/L	2.1 ± 2.3

Data are expressed as means ± SDs or N (%)

*SD* standard deviation, *ESRD* end-stage renal disease, *ADPKD* autosomal dominant polycystic kidney disease, *HTN* hypertension, *UF* ultrafiltration, *HD* haemodialysis, *OL-HDF* online haemodiafiltration, *MCO* membrane cut-off, *CRP* C-reactive protein

### Predialysis parameters and changes in the serum solutes before and after dialysis

**Fig. 1:**
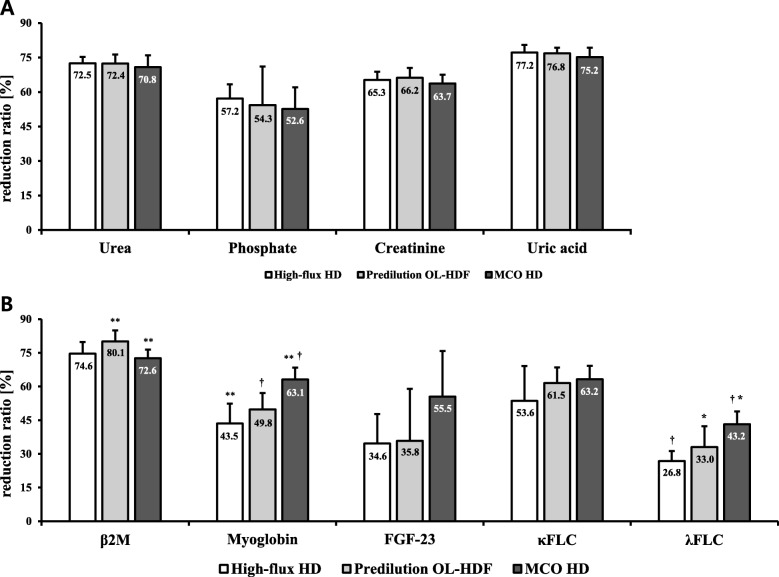
Bar graphs show reduction ratio (%) for the various uraemic toxins according to the treatment modalities. **a** Small water-soluble molecules. **b** Large (middle) molecules. Data are expressed as mean ± SDs. ^**†^*P* < 0.05/3 = 0.0167, ^*^P < 0.05 by the post hoc test using the linear mixed model with the least significant difference between two groups. SD: standard deviation; HD: haemodialysis; OL-HDF: online haemodiafiltration; MCO: membrane cut-off; β2M: β2-microglobulin; FLC: free light chain; FGF: fibroblast growth factor

There was no significant difference in the mean single-pooled Kt/V urea level among HF HD, predilution OL-HDF, and MCO HD (1.51 ± 0.14, 1.51 ± 0.18, and 1.45 ± 0.20, respectively; *P* = 0.8215). The blood cell counts and serum levels of total protein, calcium, urea, phosphate, creatinine, uric acid, β2M, myoglobin, κFLC, FGF-23, λFLC, and albumin were measured (Additional file 1). No differences were detected in the predialysis parameters, such as serum albumin, myoglobin, β2M, κFLC, λFLC, and FGF-23, among the three groups. Figure 1 and Table 3 show the RR for the small water-soluble molecules and large middle molecules during each dialysis treatment and comparison among the three dialysis modalities.

**Table 3:** Reduction ratio (%) for the various uraemic toxins and albumin according to the treatment modalities

Variables	High-flux HD (1)	Predilution OL-HDF (2)	MCO HD (3)	Overall *P*	post hoc *P*		
					(1) vs (2)	(1) vs (3)	(2) vs (3)
Urea	72.5 ± 2.8	72.4 ± 3.9	70.8 ± 5.2	0.7769	0.9686	0.4949	0.5543
Phosphate	57.2 ± 6.1	54.3 ± 16.8	52.6 ± 9.4	0.5976	0.7031	0.3275	0.8228
Creatinine	65.3 ± 3.5	66.2 ± 4.3	63.7 ± 3.8	0.5436	0.699	0.4445	0.2943
Uric acid	77.2 ± 3.3	76.8 ± 2.5	75.2 ± 4.1	0.6338	0.8099	0.3637	0.4297
β2M	74.6 ± 5.2	80.1 ± 4.9^*^	72.6 ± 3.8^*^	0.0305	0.0811	0.4466	0.0096
Myoglobin	43.5 ± 8.9^*^	49.8 ± 7.3^†^	63.1 ± 5.3^*†^	0.0005	0.2015	0.0003	0.0025
FGF-23	34.6 ± 13.1	35.8 ± 23.2	55.5 ± 20.3	0.1281	0.9192	0.0509	0.1370
κFLC	53.6 ± 15.5	61.5 ± 7.0	63.2 ± 6.0	0.3703	0.2621	0.1668	0.6542
λFLC	26.8 ± 4.4^*^	33.0 ± 9.3^a^	43.2 ± 5.7^*a^	0.0002	0.1589	< 0.0001	0.0368
Albumin	1.7 ± 3.6	2.3 ± 4.1	4.9 ± 2.8	0.2173	0.8041	0.1116	0.2227

Data are expressed as means ± standard deviations. ^*†^*P* < 0.05/3 = 0.0167, ^a^*P* < 0.05 by the post hoc test using the linear mixed model with the least significant difference between two groups

*HD* haemodialysis, *OL-HDF* online haemodiafiltration, *MCO* membrane cut-off, *β2M* β2-microglobulin, *FLC* free light chain, *FGF* fibroblast growth factor

All three dialysis treatments showed comparable RRs for the small molecular uraemic toxins (i.e. phosphate, urea, creatinine, and uric acid). MCO HD showed greater RRs for myoglobin than did standard HF HD and predilution OL-HDF (myoglobin: 63.1 ± 5.3% vs. 43.5 ± 8.9% and 49.8 ± 7.3%, *P* <  0.001). Of note, predilution OL-HDF showed the greatest RR for β2M, whereas MCO HD and HF HD showed comparable RRs for β2M (predilution OL-HDF vs. MCO HD: 80.1 ± 4.9% vs. 72.6 ± 3.8%; *P* = 0.01). Further, there was no significant difference among MCO HD, HF HD, and predilution OL-HDF in terms of the RR for FGF-23 (55.5 ± 20.3%, 34.6 ± 13.1%, and 35.8 ± 23.2%, *P* = 0.13).

### Free light chain reduction ratio and clearance during MCO HD compared to HF HD and predilution OL-HDF.

**Fig. 2:**
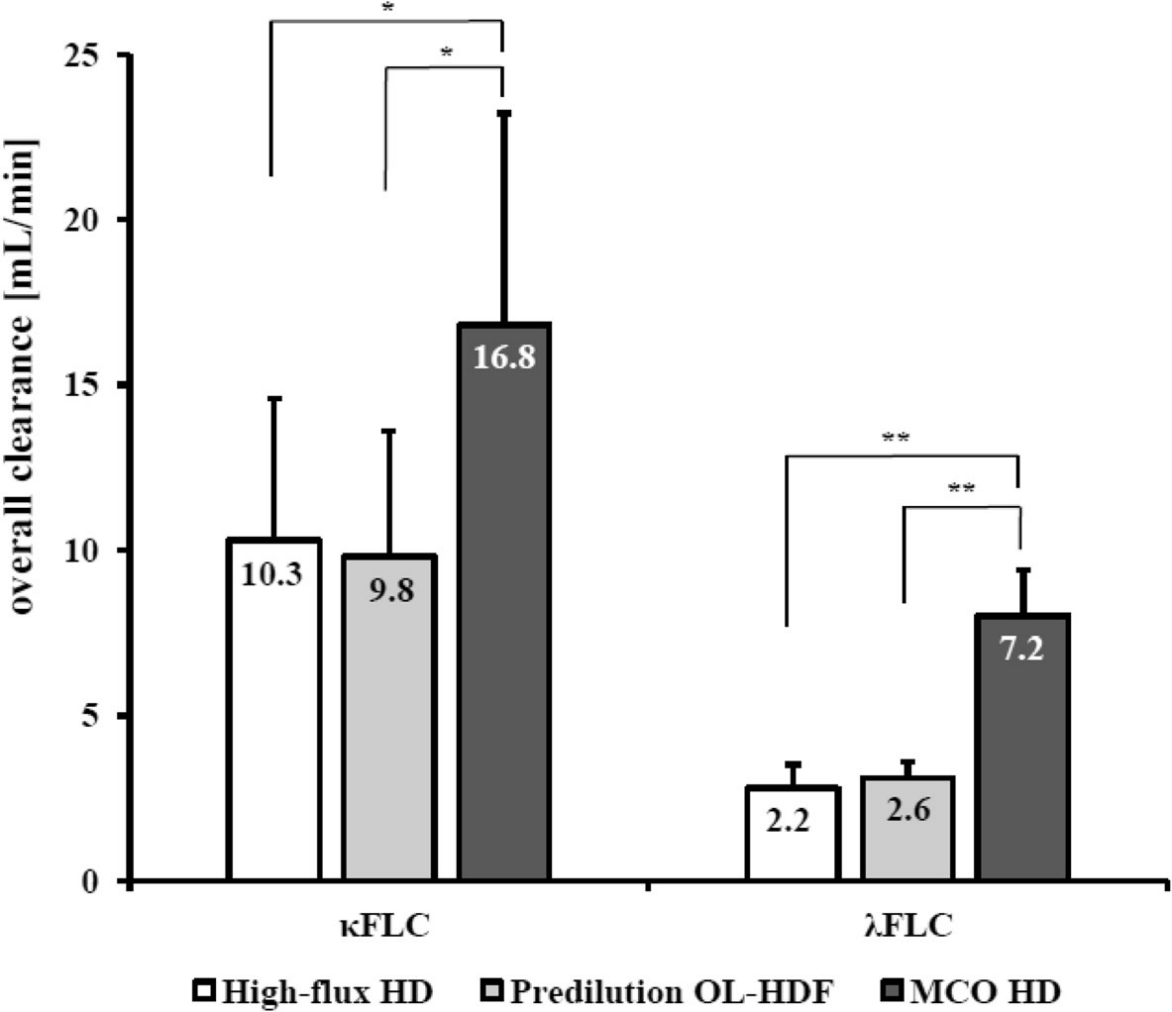
Bar graphs show FLCs clearance (mL/min) depending on treatment modalities. Data are expressed as mean ± SDs. ^**^P < 0.05/3 = 0.0167, ^*^P < 0.05 by the post hoc test using the linear mixed model with the least significant difference between two groups. SD: standard deviation; HD: haemodialysis; OL-HDF: online haemodiafiltration; MCO: membrane cut-off; FLC: free light chain

MCO HD showed significantly greater RR for λFLC compared to HF HD and OL-HDF (λFLC: 43.2 ± 5.6% vs. 26.8 ± 4.4% and 33.0 ± 9.2%, respectively; *P* < 0.001). Accordingly, clearances for λFLC was significantly greater during MCO HD compared to HF HD and OL-HDF (8.0 ± 1.4 vs. 2.8 ± 0.7 and 3.1 ± 0.5 mL/min, respectively; *P* < 0.001). No significant difference was observed for the RR for κFLC among three dialysis modalities (63.2 ± 6.0%, 53.6 ± 15.5%, and 61.5 ± 7.0%, respectively; *P* = 0.37). However, MCO HD showed almost twice the clearances for κFLC compared to HF HD or OL-HDF (16.8 ± 6.4 vs. 10.3 ± 4.3, and 9.8 ± 3.8 mL/min; *P* < 0.05) (Fig. 2).

### Albumin loss during MCO HD and predilution OL-HDF

Serum albumin levels before and after predilution OL-HDF (from 3.85 ± 0.29 to 3.76 ± 0.33 g/dL) and MCO HD (from 3.77 ± 0.30 to 3.58 ± 0.32 g/dL) showed minimal changes with no significant difference in the RR for albumin among HF HD, predilution OL-HDF, and MCO HD (1.7 ± 3.6%, 2.3 ± 4.1%, and 4.9 ± 2.8%, respectively; *P* = 0.22). In contrast, albumin leakage to the effluent by MCO HD (median 3.16 g session^− 1^, interquartile range 2.17–3.59 g session^− 1^) was greater, compared to both HF HD (median 0.06 g session^− 1^, interquartile range 0.03–0.13 g session^− 1^) and predilution OL-HDF (median 0.07 g session^− 1^, interquartile range 0.05–0.74 g session^− 1^), (*P* = 0.009) (Table 4).

**Table 4:** Albumin leakage (g session^−1^) depending on treatment modalities

Variables	High-flux HD (1)	Predilution OL-HDF (2)	MCO HD (3)	Overall *P*	post hoc *P*		
					(1) vs (2)	(1) vs (3)	(2) vs (3)
Albumin	0.06 (0.03–0.13)^a^	0.07 (0.05–0.74)^b^	3.16 (2.17–3.59)^ab^	0.009	0.249	0.028	0.028

Data are expressed as medians (Q1–Q3). ^ab^*P* < 0.05 by the post hoc test using the linear mixed model with the least significant difference between two groups

*HD* haemodialysis, *OL-HDF* online haemodiafiltration, *MCO* membrane cut-off

### Safety

During the study, there were no episodes of intradialytic hypotension and no clinically significant complications reported.

## Discussion

The primary aim of this study was to investigate the efficacy of three different HD modalities commonly used in clinical practice: HF HD, predilution OL-HDF, and the newly introduced MCO HD in removing middle to large molecular-weight uraemic solutes at relatively low BFR. We found somewhat varying results regarding the large middle molecular reduction properties among the three dialysis modalities. MCO HD showed the greatest RRs for myoglobin and λFLC, and the gap was markedly great in relation to those in HF HD and predilution OL-HDF. In contrast, no significant difference was observed for the RR for κFLC among three dialysis modalities. Meanwhile, MCO HD showed highest overall clearance for FLCs. Predilution OL-HDF showed the greatest RR for β2M, and MCO HD unexpectedly showed the lowest RR for such. The extent of middle molecular reduction was not predicted by its molecular weight, as the extent of reduction depended not only on the size of the molecule but also on other physiochemical properties, such as charge, hydrophilicity, or membrane binding [17]. The overall RRs for the small water-soluble molecules, such as phosphate, urea, creatinine, and uric acid, were comparable among the three dialysis modalities.

Recently, Kirsch et al. demonstrated that HD using MCO membranes at a BFR of 300–400 mL/min efficiently remove large middle molecules [12]. Our study further demonstrate that even at lower BFRs (250 mL/min), MCO HD can achieve efficient RRs for large middle molecules and clearance of FLCs compared to HF HD or predilution OL-HDF. Notably, the reduced efficiency in both diffusive and convective solute removal in predilution OL-HDF may account for the observed lower RR for large middle molecules and FLC clearance in our study. However, the mean convection volume achieved during our predilution OL-HDF treatments was 49.9 L/session that is larger than the mean convection volume that has been shown to confer survival advantage for both all-cause mortality and CV mortality in Japanese patients with ESRD on predilution OL-HDF [18]. This is convection volume comparable with that recommended by recent clinical studies on postdilution OL-HDF [19] and indicate that our OL-HDF treatment was assessed at its proper capacity.

The RR of FLCs and myoglobin in our study using MCO HD are similar to those of MCO AA reported by Kirsch et al. (κFLC: 66.3 to 72.9% and λFLC: 42.5 to 52.71%, myoglobin 63.1 to 67.9%). This MCO AA dialyzer has the most similar membrane characteristics to the Theranova 400® used in our study based on the manufacturer’s information. Moreover, the clearances for FLCs observed in our study using MCO HD are also close to those of Kirsch et al. (κFLC 26.2 to 35.0 mL/min and λFLC 8.5 to 10.0 mL/min) and the small differences observed could be potentially attributed to variations in dialysis parameters. Interestingly, despite similar RR for κFLC among three dialysis modalities, κFLC clearance was much higher with MCO HD. This finding suggests that MCO HD may provide greater removal of middle molecules.

Predilution OL-HDF showed a greater RR for β2M than did MCO HD, and HF HD and MCO HD achieved comparable RRs for such. It is well known that higher BFR, larger surface size, and larger convection volume increase β2M clearance in HF HD or HDF. Dialyser membrane characteristics may also contribute to RR and Rexeed-21A® demonstrates good clearance for β2M in both HD and HDF treatments [20]. The positive effects of a high convection volume and a larger membrane surface area are well demonstrated by the better β2M RR achieved in OL-HDF. The extent of middle molecular reduction was not predicted by its molecular weight, as the extent of reduction depended not only on the size of the molecule but also on other physiochemical properties, such as charge, hydrophilicity, or membrane binding [17]. It may be difficult to compare our study data directly with previous studies in the literature because of variability in dialysis BFRs, dialyzer characteristics, and convection volumes. Notably, patients enrolled in the present study could not tolerate high BFR that is prerequisite for postdilution HDF with high substitution volume. Therefore, the BFR used for all dialysis modalities in our study was fixed at 250 mL/min to mimic actual clinical practice settings where HD patients with poor vascular access cannot tolerate higher BFR. Among Korean HD patients enrolled in Clinical Research Center registry for ESRD, median value of BFR was 250 mL/min and average BFR for Japanese HD patients treated with predilution OL-HDF is 230.8 ± 42.9 mL/min. Therefore, evaluation of MCO HD at a low BFR can be meaningful for many Asian patients as well as elderly HD patients with poor vascular access.

In maintenance HD patients, increased levels of circulating FGF-23 are independently associated with CV events and mortality [21]. Particularly, increased FGF-23 levels in patients with CKD are clinically relevant to CV mortality [22] by inducing left ventricular hypertrophy [23, 24], arterial stiffness combined with endothelial dysfunction [25], and vascular calcification [26]. Owing to these untoward effects of FGF-23 per se, potential therapeutic options to reduce the levels of FGF-23 in HD patients are warranted. Our study demonstrated a tendency for greater removal of FGF-23 with MCO HD than with HF HD, as shown by the greater RR (55.5 ± 20.3% vs. 34.6 ± 13.1%; *P* = 0.0509). Notably, predilution OL-HDF failed to show significantly greater RRs for FGF-23 in contrast to HF HD (35.7 ± 23.2% vs. 34.6 ± 13.1%). This is in contrast to previously reported results where OL-HDF demonstrated improved removal of FGF-23 when compared with that of HF HD [27]. Compared with the other uraemic middle molecules studied herein, FGF-23 showed a large range of removal values, which strongly suggests an intra-individual variability between different circulating forms of FGF-23 [28] and phosphocalcic metabolic status [27]. Different forms of FGF-23 with different molecular weights, such as N-terminal (18.0 kD) or C-terminal (12.0 kD) fragments, as well as intact FGF-23, are circulating in the blood of patients [28]. The second-generation FGF-23 (C-terminal) ELISA kit used in our study measures both human intact FGF-23 and C-terminal fragments of FGF-23. Accordingly, similar to our study findings, the comparison between HF HD and OL-HDF in the study by Patrier et al. showed a large range of RR for FGF-23 (5.3–74.3% vs. 26.6–75.9%) [27]. Nevertheless, our finding suggests tendency for more intense elimination of FGF-23 by MCO HD compared to HF HD.

Loss of albumin through efflux via high convection volumes is considered to be one of the disadvantages of OL-HDF as well as MCO HD [29–31]. In our study, RR for albumin with MCO HD was not significantly higher compared to other two treatment modalities. However, MCO HD showed greater loss of albumin during a single HD treatment, which is comparable to results of Kirsch’s study (2.9 to 3.2 g session^− 1^). Recent studies reported no significant decrease in serum albumin levels over 6 to 12-month period of treatment with MCO dialyzer. Long-term studies on MCO HD using low BFR are needed to assess whether there are any change in serum albumin levels [32, 33].

There are several limitations to our study. First, only a small number of male patients were enrolled and the RR for the uraemic toxins were derived from a single treatment. Second, RR does not accurately evaluate removal of large uraemic toxins. Post-dialysis rebound of middle molecules such as β2M is substantial and therefore results in RR measurement to overestimate β2M clearance when compared to clearance determined directly across the dialyzer [34]. This rebound in solutes probably result from redistribution of large solutes from interstitium to the plasma after dialysis treatment [35]. However, RR of FLCs from MCO HD are in line with those of clearance FLC measurement in our study. Therefore, RR may still function as an incomplete marker of middle molecule removal when direct measurements are difficult. Third, we did not elute each membrane to take into account any potential adsorption of middle molecules to the dialyzer membranes. However, other studies have demonstrated that adsorption of middle molecules to dialyzer membranes is not a major factor of the overall removal of large middle molecules [12]. Lastly, we did not conduct any follow-up and did not investigate the long-term effects on the serum levels of the middle molecules. Recent studies with MCO HD treatment duration of 6 to 12 months reported largely negative results on plasma levels of middle molecules. But, both studies were limited by retrospective or observational nature of the study and lacked control for residual renal function. Large randomized controlled trials of longer duration are needed to make any firm conclusions.

## Conclusions

In conclusion, MCO HD at low BFR showed significantly greater reduction of large middle molecules and clearances for FLCs than did conventional HD and OL-HDF. This was possible without the need for large convection volumes or high BFR. This would pose as an advantage not only for elderly HD patients with poor vascular access but also for those without access to OL-HDF.

## Supplementary information

**Additional file 1. **Pre- and postdialysis serum levels of the various parameters among the different treatment modalities. Data are expressed as means ± SDs or medians (Q1–Q3). The postdialysis levels of the large middle molecules and albumin were corrected for haemoconcentration. The *P* was given for the overall result of pre- and postdialysis between the three groups and < 0.05 was considered statistically significant. SD: standard deviation; NS, not significant; HD: haemodialysis; OL-HDF: online haemodiafiltration; MCO: membrane cut-off; β2M: β2-microglobulin; FLC: free light chain; FGF: fibroblast growth factor.

## Data Availability

The datasets that support the findings of the current study are available from the corresponding author on reasonable request.

## Abbreviations

BFR: Blood flow rate
CV: Cardiovascular
CVD: Cardiovascular disease
ESRD: End-stage renal disease
FGF: Fibroblast growth factor
FLC: Free light chain
HD: Haemodialysis
HDF: Haemodiafiltration
HF: High-flux
IL: Interleukin
MCO: Medium cut-off
OL-HDF: Online haemodiafiltration
RR: Reduction ratio
β2M: β2-microglobulin

## References

[1] Sirich TL, Meyer TW (2018). Intensive hemodialysis fails to reduce plasma levels of uremic solutes. Clin J Am Soc Nephrol.

[2] Fraser SDS, Fenton A, Harris S, Shardlow A, Liabeuf S, Massy ZA, Burmeister A, Hutchison CA, Landray M, Emberson J (2017). The Association of Serum Free Light Chains with Mortality and Progression to end-stage renal disease in chronic kidney disease: systematic review and individual patient data meta-analysis. Mayo Clin Proc.

[3] Wolley MJ, Hutchison CA (2018). Large uremic toxins: an unsolved problem in end-stage kidney disease. Nephrol Dial Transplant.

[4] Canaud B, Bragg-Gresham JL, Marshall MR, Desmeules S, Gillespie BW, Depner T, Klassen P, Port FK (2006). Mortality risk for patients receiving hemodiafiltration versus hemodialysis: European results from the DOPPS. Kidney Int.

[5] Grooteman MP, van den Dorpel MA, Bots ML, Penne EL, van der Weerd NC, Mazairac AH, den Hoedt CH, van der Tweel I, Levesque R, Nube MJ (2012). Effect of online hemodiafiltration on all-cause mortality and cardiovascular outcomes. J Am Soc Nephrol.

[6] Ok E, Asci G, Toz H, Ok ES, Kircelli F, Yilmaz M, Hur E, Demirci MS, Demirci C, Duman S (2013). Mortality and cardiovascular events in online haemodiafiltration (OL-HDF) compared with high-flux dialysis: results from the Turkish OL-HDF study. Nephrol Dial Transplant.

[7] Morena M, Jaussent A, Chalabi L, Leray-Moragues H, Chenine L, Debure A, Thibaudin D, Azzouz L, Patrier L, Maurice F (2017). Treatment tolerance and patient-reported outcomes favor online hemodiafiltration compared to high-flux hemodialysis in the elderly. Kidney Int.

[8] Vanholder R, Laecke SV, Verbeke F, Glorieux G, Biesen WV (2008). Uraemic toxins and cardiovascular disease: in vitro research versus clinical outcome studies. NDT Plus.

[9] Schiffl H (2019). Online hemodiafiltration and mortality risk in end-stage renal disease patients: a critical appraisal of current evidence. Kidney Res Clin Pract.

[10] Wolley M, Jardine M, Hutchison CA (2018). Exploring the clinical relevance of providing increased removal of large middle molecules. Clin J Am Soc Nephrol.

[11] Boschetti-de-Fierro A, Voigt M, Storr M, Krause B (2015). MCO membranes: enhanced selectivity in high-flux class. Sci Rep.

[12] Kirsch AH, Lyko R, Nilsson LG, Beck W, Amdahl M, Lechner P, Schneider A, Wanner C, Rosenkranz AR, Krieter DH (2017). Performance of hemodialysis with novel medium cut-off dialyzers. Nephrol Dial Transplant.

[13] Garcia-Prieto A, Vega A, Linares T, Abad S, Macias N, Aragoncillo I, Torres E, Hernandez A, Barbieri D, Luno J (2018). Evaluation of the efficacy of a medium cut-off dialyser and comparison with other high-flux dialysers in conventional haemodialysis and online haemodiafiltration. Clin Kidney J.

[14] Bergstrom J, Wehle B (1987). No change in corrected beta 2-microglobulin concentration after cuprophane haemodialysis. Lancet.

[15] Schneditz D, Putz-Bankuti C, Ribitsch W, Schilcher G (2012). Correction of plasma concentrations for effects of hemoconcentration or hemodilution. ASAIO J.

[16] Basile C, Libutti P, Di Turo AL, Casino FG, Vernaglione L, Tundo S, Maselli P, De Nicolo EV, Ceci E, Teutonico A (2011). Removal of uraemic retention solutes in standard bicarbonate haemodialysis and long-hour slow-flow bicarbonate haemodialysis. Nephrol Dial Transplant.

[17] Maduell F, Navarro V, Cruz MC, Torregrosa E, Garcia D, Simon V, Ferrero JA (2002). Osteocalcin and myoglobin removal in on-line hemodiafiltration versus low- and high-flux hemodialysis. Am J Kidney Dis.

[18] Masakane I, Kikuchi K, Kawanishi H (2017). Evidence for the clinical advantages of Predilution on-line Hemodiafiltration. Contrib Nephrol.

[19] Maduell F, Moreso F, Pons M, Ramos R, Mora-Macia J, Carreras J, Soler J, Torres F, Campistol JM, Martinez-Castelao A (2013). High-efficiency postdilution online hemodiafiltration reduces all-cause mortality in hemodialysis patients. J Am Soc Nephrol.

[20] Brendolan A, Nalesso F, Fortunato A, Crepaldi C, De Cal M, Cazzavillan S, Cruz D, Techawathanawanna N, Ronco C (2005). Dialytic performance evaluation of Rexeed: a new polysulfone-based dialyzer with improved flow distributions. Int J Artif Organs.

[21] Gutierrez OM, Mannstadt M, Isakova T, Rauh-Hain JA, Tamez H, Shah A, Smith K, Lee H, Thadhani R, Juppner H (2008). Fibroblast growth factor 23 and mortality among patients undergoing hemodialysis. N Engl J Med.

[22] Parker BD, Schurgers LJ, Brandenburg VM, Christenson RH, Vermeer C, Ketteler M, Shlipak MG, Whooley MA, Ix JH (2010). The associations of fibroblast growth factor 23 and uncarboxylated matrix Gla protein with mortality in coronary artery disease: the heart and soul study. Ann Intern Med.

[23] Gutierrez OM, Januzzi JL, Isakova T, Laliberte K, Smith K, Collerone G, Sarwar A, Hoffmann U, Coglianese E, Christenson R (2009). Fibroblast growth factor 23 and left ventricular hypertrophy in chronic kidney disease. Circulation.

[24] Faul C, Amaral AP, Oskouei B, Hu MC, Sloan A, Isakova T, Gutierrez OM, Aguillon-Prada R, Lincoln J, Hare JM (2011). FGF23 induces left ventricular hypertrophy. J Clin Invest.

[25] Mirza MA, Larsson A, Lind L, Larsson TE (2009). Circulating fibroblast growth factor-23 is associated with vascular dysfunction in the community. Atherosclerosis.

[26] Khan AM, Chirinos JA, Litt H, Yang W, Rosas SE (2012). FGF-23 and the progression of coronary arterial calcification in patients new to dialysis. Clin J Am Soc Nephrol.

[27] Patrier L, Dupuy AM, Granger Vallee A, Chalabi L, Morena M, Canaud B, Cristol JP (2013). FGF-23 removal is improved by on-line high-efficiency hemodiafiltration compared to conventional high flux hemodialysis. J Nephrol.

[28] Smith ER (2014). The use of fibroblast growth factor 23 testing in patients with kidney disease. Clin J Am Soc Nephrol.

[29] den Hoedt CH, Bots ML, Grooteman MP, van der Weerd NC, Mazairac AH, Penne EL, Levesque R, ter Wee PM, Nube MJ, Blankestijn PJ (2014). Online hemodiafiltration reduces systemic inflammation compared to low-flux hemodialysis. Kidney Int.

[30] Movilli E, Camerini C, Gaggia P, Zubani R, Feller P, Salviani C, Facchini A, Cancarini G (2015). Total convection affects serum beta2 microglobulin and C-reactive protein but not erythropoietin requirement following post-Dilutional Hemodiafiltration. Am J Nephrol.

[31] Jean G, Hurot JM, Deleaval P, Mayor B, Lorriaux C (2015). Online-haemodiafiltration vs conventional haemodialysis: a cross-over study. BMC Nephrol.

[32] Cho NJ, Park S, Islam MI, Song HY, Lee EY, Gil HW (2019). Long-term effect of medium cut-off dialyzer on middle uremic toxins and cell-free hemoglobin. PLoS One.

[33] Belmouaz M, Diolez J, Bauwens M, Duthe F, Ecotiere L, Desport E, Bridoux F (2018). Comparison of hemodialysis with medium cut-off dialyzer and on-line hemodiafiltration on the removal of small and middle-sized molecules. Clin Nephrol.

[34] Leypoldt JK, Cheung AK, Deeter RB (1999). Rebound kinetics of beta2-microglobulin after hemodialysis. Kidney Int.

[35] Ward RA, Greene T, Hartmann B, Samtleben W (2006). Resistance to intercompartmental mass transfer limits beta2-microglobulin removal by post-dilution hemodiafiltration. Kidney Int.

