# Supplementary material for: Removal of large middle molecules via haemodialysis with medium cut-off membranes at lower blood flow rates: an observational prospective study
Source: BMC Nephrol. 2019 Dec 31;21:2. doi: 10.1186/s12882-019-1669-3 (PMC6937993; doi:10.1186/s12882-019-1669-3)
Supplement: Supplementary file 1 — Additional file 1. Pre- and postdialysis serum levels of the various parameters among the different treatment modalities. Data are expressed as means ± SDs or medians (Q1–Q3). The postdialysis levels of the large middle molecules and albumin were corrected for haemoconcentration. The P was given for the overall result of pre- and postdialysis between the three groups and < 0.05 was considered statistically significant. SD: standard deviation; NS, not significant; HD: haemodialysis; OL-HDF: online haemodiafiltration; MCO: membrane cut-off; β2M: β2-microglobulin; FLC: free light chain; FGF: fibroblast growth factor. [file 12882_2019_1669_MOESM1_ESM.pdf]

		White blood cell count, 10^3/µL	Haemoglobin level, g/dL	Haematocrit level, %	Platelet level, 10^3/µL	Calcium level, mg/dL
**High-flux HD**	Pre	4.8 ± 0.9	10.5 ± 0.8	31.9 ± 2.7	151.1 ± 39.1	8.6 ± 0.9
	Post	4.9 ± 0.9	11.6 ± 1.1	35.0 ± 3.7	167.3 ± 45.1	9.8 ± 0.8
**Predilution OL-HDF**	Pre	5.2 ± 1.8	10.8 ± 0.9	32.4 ± 2.6	156.5 ± 33.2	8.5 ± 0.6
	Post	5.2 ± 1.9	12.1 ± 1.2	36.2 ± 4.2	170.7 ± 49.0	9.4 ± 0.7
**MCO HD**	Pre	5.4 ± 2.0	10.8 ± 1.0	32.2 ± 3.1	170.8 ± 59.5	8.6 ± 0.7
	Post	5.4 ± 2.0	12.5 ± 1.2	36.5 ± 4.0	185.3 ± 60.4	9.7 ± 0.5
P	Pre	NS	NS	NS	NS	NS
	Post	NS	NS	NS	NS	NS

		Urea level, mg/dL	Phosphate level, mg/dL	Creatinine level, mg/dL	Uric acid level, mg/dL	Albumin level, g/dL
**High-flux HD**	Pre	55.9 ± 12.0	4.9 ± 0.9	9.5 ± 2.0	6.5 ± 1.0	3.83 ± 0.34
	Post	15.4 ± 3.7	2.1 ± 0.6	3.3 ± 0.9	1.5 ± 0.3	3.77 ± 0.33
**Predilution OL-HDF**	Pre	56.8 ± 14.6	4.8 ± 1.3	9.9 ± 2.1	6.5 ± 0.8	3.85 ± 0.29
	Post	15.4 ± 3.1	2.1 ± 0.6	3.4 ± 0.9	1.5 ± 0.3	3.76 ± 0.33
**MCO HD**	Pre	55.0 ± 10.6	4.6 ± 1.1	9.5 ± 2.0	6.3 ± 0.4	3.77 ± 0.30
	Post	15.9 ± 3.4	2.2 ± 0.5	3.5 ± 0.9	1.6 ± 0.3	3.58 ± 0.32
P	Pre	NS	NS	NS	NS	NS
	Post	NS	NS	NS	NS	NS

		β2M level, mcg/mL	Myoglobin level, mcg/L	FGF-23 level, pg/mL	κFLC level, mg/L	λFLC level, mg/L
**High-flux HD**	Pre	24.8 ± 5.5	208.6 ± 53.6	996.8 ± 471.1	166.6 (144.4–228.9)	136.1 ± 17.5
	Post	6.1 ± 0.8	116.4 ± 30.5	750.9 ± 390.3	87.6 (49.9–149.2)	100.3 ± 18.4
**Predilution OL-HDF**	Pre	22.2 ± 8.2	210.1 ± 61.2	899.4 ± 515.9	155.4 (130.2–208.2)	140.0 ± 13.8
	Post	4.1 ± 0.7	106.5 ± 40.5	635.7 ± 391.8	66.9 (43.1–96.1)	93.4 ± 12.5
**MCO HD**	Pre	19.4 ± 6.2	232.6 ± 93.5	819.9 ± 589.2	121.0 (163.8–269.0)	135.7 ± 10.4
	Post	5.2 ± 1.3	84.1 ± 34.3	533.2 ± 410.1	58.4 (46.7–118.7)	77.1 ± 9.3
P	Pre	NS	0.8547	NS	NS	NS
	Post	0.0013	0.2535	NS	0.0420	0.0158
